# *Candida* in Lower Respiratory Tract Increases the Frequency of Acute Exacerbation of Chronic Obstructive Pulmonary Disease: A Retrospective Case-Control Study

**DOI:** 10.3389/fcimb.2020.538005

**Published:** 2020-09-30

**Authors:** Yi-hui Zuo, Wei-qin Wang, Qi-jian Chen, Bin Liu, Feng-ying Zhang, Xiao-yan Jin, Jing-qing Hang, Hua-yin Li, Zhi-yao Bao, Zhi-jun Jie, Gui-fang Wang, Xi-wen Gao, He Sun, Jin-fu Xu, Jing Zhang, Jie-ming Qu

**Affiliations:** ^1^Department of Pulmonary and Critical Care Medicine, Zhongshan Hospital, Fudan University, Shanghai, China; ^2^Department of Respiratory Medicine, Tongren Hospital, School of Medicine, Shanghai Jiao Tong University, Shanghai, China; ^3^Department of Respiratory Medicine, School of Medicine, Ruijin Hospital, Shanghai Jiao Tong University, Shanghai, China; ^4^Department of Respiratory Medicine, School of Medicine, Ruijin North Hospital, Shanghai Jiao Tong University, Shanghai, China; ^5^Department of Respiratory Medicine, School of Medicine, Renji Hospital, Shanghai Jiao Tong University, Shanghai, China; ^6^Department of Respiratory Medicine, Shanghai Putuo District People's Hospital, Shanghai, China; ^7^Department of Respiratory Medicine, Shanghai Fifth' Peoples Hospital, Fudan University, Shanghai, China; ^8^Department of Respiratory Medicine, Huashan Hospital, Fudan University, Shanghai, China; ^9^Department of Respiratory Medicine, Central Hospital of Minhang District, Shanghai, Fudan University, Shanghai, China; ^10^Department of Respiratory Medicine, Shanghai General Hospital, School of Medicine, Shanghai Jiao Tong University, Shanghai, China; ^11^Department of Respiratory Medicine, Shanghai Pulmonary Hospital, Tongji University, Shanghai, China

**Keywords:** acute exacerbation of chronic obstructive pulmonary disease (AECOPD), prognosis, *Candida*, recurrence of AECOPD, mortality

## Abstract

**Objective:** To explore impact of *Candida* on the acute exacerbation of chronic obstructive pulmonary disease (AECOPD) outcome.

**Methods:** A retrospective, multi-center, case-control study was performed. Patients hospitalized for AECOPD in 25 centers during Jan 2011–Dec 2016 were enrolled. Data were collected, including demographic information, conditions during the stable phase of COPD, clinical characteristics of AECOPD, and follow-up information within 1 year after discharge. Univariate analysis and binary logistic regression were applied, and *p* < 0.05 was regarded as significant.

**Results:** Totally 1,103 patients were analyzed, with 644 lower respiratory airway (LTR) *Candida* positive cases and 459 *Candida* negative controls. Long-term prognosis was significantly different between *Candida* positive and negative group, including the recurrent AECOPD within 180 days (75.5 vs. 6.6%, *p* < 0.001) and mortality within 1 year (6.9 vs. 0.4%, *p* < 0.001). Univariate logistic analysis showed that LTR *Candida* isolation was related to higher recurrence rate of AECOPD within 180 days and mortality within 1 year. Binary logistic regression analysis demonstrated that LTR *Candida* isolation was independently associated with recurrence of AECOPD within 180 days.

**Conclusions:** LTR *Candida* isolation was associated with worse long-term prognosis of AECOPD and independently related to higher risks of recurrent AECOPD within 180 days.

## Introduction

Chronic obstructive pulmonary disease (COPD) is one of the most common chronic diseases with high morbidity and mortality. According to the 2019 global initiative for COPD (GOLD) report, COPD is the fourth leading cause of death in the world. It is expected to rise to the third place by 2020, and more than 3 million people died of COPD in 2012, accounting for 6% of the total number of deaths in the world (GOLD, 2019). According to the Global Burden of Disease Study data, COPD is the third leading cause of death in China, and the mortality rate is increasing (Wang, 2017). Acute exacerbation of COPD (AECOPD) is the most important acute event in the disease course of COPD, leading to disease progression and poor outcome. It has been reported that every patient with COPD suffered from 1.5 exacerbations a year on average (Wedzicha and Seemungal, 2007). The most common causes of AECOPD were viruses and bacterial infections (Aaron, 2014; Cai et al., 2014). Co-infection of viruses and bacteria has been detected in 25% of exacerbations (Sethi and Murphy, 2008).

*Candida* is one of the normal floras of human body, and is also the most common conditional fungal pathogen. Invasive *Candida* infection (ICI) occupies the first place in invasive fungal disease (IFD) (Pfaller and Diekema, 2007). At the same time, *Candida* is commonly colonized in lower respiratory airway (LTR). It was found that the main risk factors for high *Candida* isolation rates in LTR were COPD, smoking, tuberculosis, malnutrition, malignant tumors, diabetes, HIV infection, and long-term use of antibiotics. Among them, the LTR isolation rate of *Candida* in COPD patients was the highest (76.65%) (Jha et al., 2006), particularly in elder patients (≥60) with persistent disease progression, recurrent infection, long-term repeated use of broad-spectrum antibiotics and systemic glucocorticoids application, mechanical ventilation (MV) and invasive procedures, or along with other chronic underlying diseases such as diabetes (Liu and Su-Rong, 2015). Broad-spectrum antibiotics and glucocorticoids are widely used in the treatment of hospitalized AECOPD, which contribute to the high colonization rate of *Candida albicans* in these patients (Bassetti et al., 2013; De Rosa et al., 2015; Hii et al., 2015). To our knowledge, studies focused on the impact of *Candida* colonization on respiratory disease were mainly about ventilator-associated pneumonia (VAP) and intensive care unit (ICU)-acquired pneumonia (Williamson et al., 2011; Dong-xing et al., 2016; Terraneo et al., 2016), while impact of *Candida* colonization on the prognosis of AECOPD was rarely reported. The object of our study was to explore the impact of LTR *Candida* isolation on the short-term and long-term prognosis of hospitalized AECOPD, which might have important clinical significance for the prevention and treatment of COPD patients.

## Materials and Methods

### Study Design and Patient Population

A retrospective, multicenter, case-control study was performed to investigate the relationship between *Candida* isolation in the respiratory tract and the prognosis of hospitalized AECOPD. From January 1st, 2011 to December 31st, 2016, patients hospitalized for AECOPD in 25 hospitals in Shanghai were collected. Only the hospitals where *Candida* culture of LTR samples were routinely performed and followed during hospitalization for admitted patients with AECOPD were involved into the current study. LTR samples included sputum, endotracheal suction, bronchoscopic flushing and lavage fluid.

The inclusion criteria were as follows: (1). Patients older than 18 years old, hospitalized for AECOPD who met the diagnostic criteria of COPD and acute exacerbation in 2017 GOLD report. Definition of acute exacerbation is the process of acute onset, which is characterized by deterioration of respiratory symptoms, beyond the scope of daily variation, and the need to change drug treatment regimens. (2) Patients with 1 year follow-up data. Those who died <1 year after hospitalization were also involved.

The exclusion criteria were the existence of any of the following: (1). Patients under 18 years old or with mental illness who were not capable of cooperating with access to clinical data; (2). Patients with lung cancer, bronchiectasis, lung abscess, interstitial pulmonary diseases; (3). Positive results of *Candida* smears or *Candida* culture in blood, pleural effusion or urine samples.

### Data Collection

According to the smears or culture results of respiratory tract specimens, all the participants were divided into *Candida* positive group and *Candida* negative group.

The collected clinical variables are listed in Table 1. The collected data were input into the Epidata system (The EpiData Association, Odense, Denmark) by one investigator, and then checked by another. Any inconsistent data found would be checked and corrected according to the original case report forms.

**Table 1:** Data collected in current study.

**Contents**
Age
Gender
Conditions GOLD grades
mMRC score
CAT score
PFTs
Complications
Fever
Maximum value of blood routine test
CRP
PCT
ESR
Blood gas analysis
Needs for oxygen inhalation
CT examination^‡^
Length of hospital stay
Hospitalization expense
ICU admission
Length of ICU stay
Time to the first re-exacerbation within 1 year^†^
Recurrent AECOPD within 180 days
Number of recurrences of AECOPD within 1 year
Mortality within 1 year
Recurrent AECOPD within 180 days
Mortality within 1 year

† For patients without re-exacerbation within 1 year, time was recorded as 365 days.

‡ Including patchy, consolidation, and multi-lobe lesions.

*COPD, chronic obstructive pulmonary disease; AECOPD, acute exacerbation of chronic obstructive pulmonary disease; CAT, COPD assessment test; mMRC, modified British research council; PFTs, pulmonary function tests; CRP, C-reaction protein; PCT, procalcitonin; ESR, erythrocyte sedimentation rate; CT, computed tomography; ICU, intensive care unit*.

The first clinical outcome is recurrent AECOPD within 180 days, and the second clinical outcome is mortality within 1 year.

### Ethical Approval

The ethics committee of Ruijin Hospital approved the study (Number: 2017-108) according to the principles of the Declaration of Helsinki. Informed consent was waived because the study was retrospective. The study was registered on Chinese Clinical Trial Registry (Registration Number: ChiCTR-RRC-17014078).

### Data Analysis

All the data were statistically analyzed by SPSS 22 system (SPSS Inc, Chicago, IL).

Firstly, comparison of conditions during the stable phase of COPD between *Candida* positive and negative groups was conducted. One-sample Kolmogorov-Smirnoff test was used to evaluate the normality of continuous variables. The continuous variables with normality were expressed as means with standard deviations and assessed for differences by Student *t*-test. For non-normal variables, medians are presented with interquartile ranges (IQR), and the Mann-Whitney *U*-test was performed when comparing two groups. The categorical variables were described as rate (%) or percentage (%), and Chi square tests or two-tailed Fisher's exact test were performed for comparisons.

Secondly, patients were separately grouped according to relapse within 180 days or death within 1 year, to identify risk factors associated with the prognosis of AECOPD. Univariate logistic regression was applied in the analysis of risk factors related to prognosis of AECOPD. Variables significant in univariate analysis (*P* < 0.05) were further evaluated in the binary logistic regression analysis. All reported *p*-values are 2-tailed, and *P* < 0.05 was taken as significant.

For missing data, patients without outcomes were withdrawn; and risk factors were deleted when missing data was over 20%.

## Results

**Figure 1:**
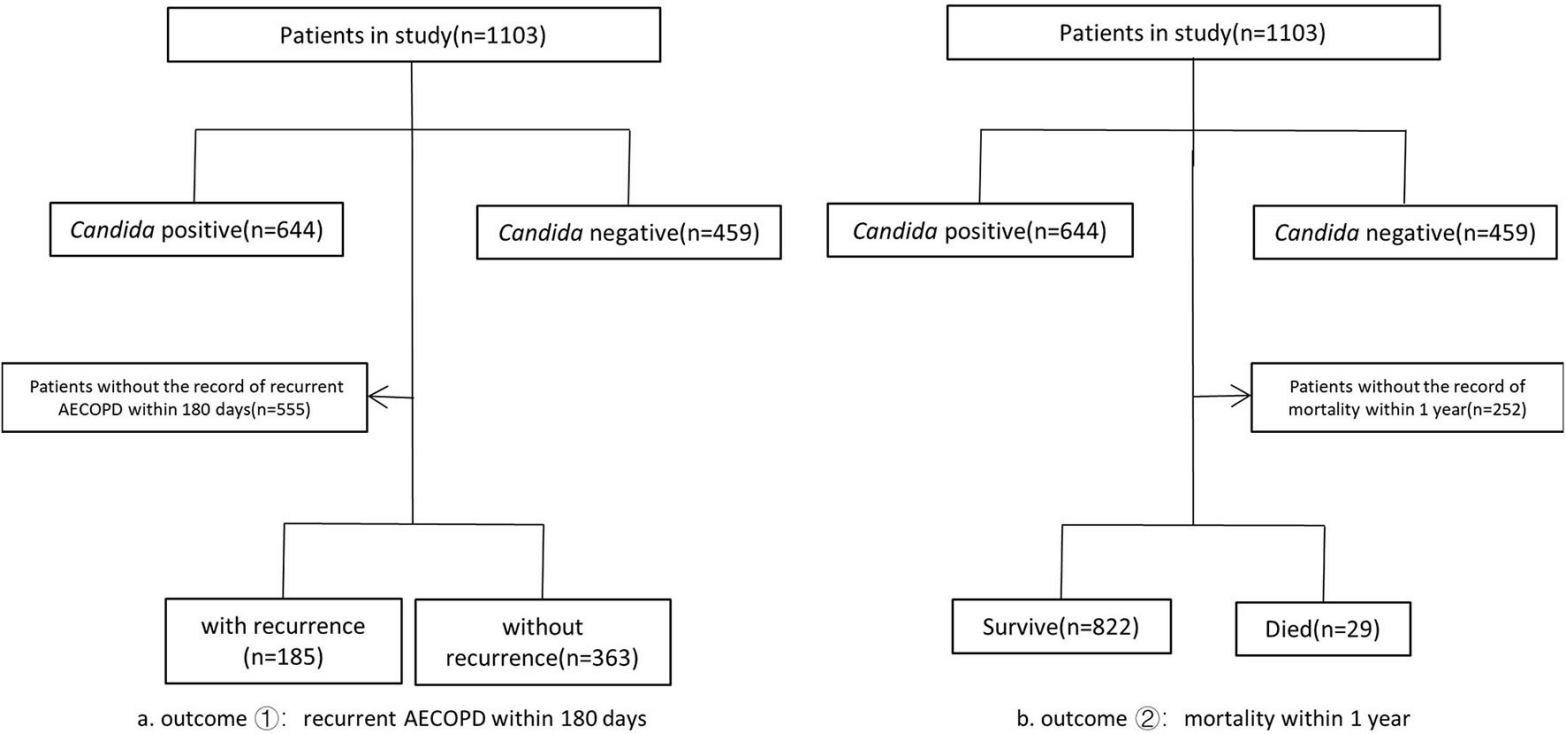
Study population flow chart. The total number of patients enrolled was 1,103. Patients were divided into Candida positive group(*n* = 644) and Candida negative group(*n* = 459) according to the smears or culture results of respiratory tract specimens. Death within 1 year and recurrent AECOPD within 180 days were two outcomes analyzed separately in our study. We withdrew patients without the record of death within 1 year (*n* = 252) and patients without the record of recurrent AECOPD within 180 days(*n* = 555) separately, and the remained patients were divided into two groups according to the outcomes, respectively.

A total of 1,103 patients were enrolled in this study, including *Candida* negative controls (*n* = 459) and *Candida* positive cases (*n* = 644). There were 944 male patients and 159 female patients. The flowchart was showed in Figure 1.

### Patients With Severe Symptoms or Complications Tend to Have Positive LTR *Candida*

We compared the conditions during the stable phase of AECOPD between *Candida* positive and *Candida* negative group. The median age of *Candida* positive group was 78 (69, 83) years old, including 556 male patients and 88 female patients, and the median age of *Candida* negative group was 78 (69, 84) years old, including 388 male patients and 71 female patients. No difference in age and sex ratio between the two groups was found.

Data analysis showed that CAT score in *Candida* positive group was slightly higher than that of *Candida* negative group (24 (19, 30) vs. 22 (15, 17), *P* < 0.001). The proportion of patients with mMRC grade 3–4 was also higher in the *Candida* positive group (71.5 vs. 58.3%, *P* < 0.001), which indicated that COPD patients with severe symptoms were more likely to be *Candida* positive.

However, no significant difference was observed in the value of forced expiratory volume in 1 s (FEV1) between the two groups during the stable phase, suggesting that severity of airflow limitation may not related to the LTR *Candida*. Both the FEV1/FVC% value of *Candida* positive group and *Candida* negative group were lower than 70%, which confirmed the diagnosis of COPD.

In terms of complications during the stable phase of COPD, the *Candida* positive group had a higher proportion of left ventricular dysfunction and obstructive sleep apnea syndrome (OSAS) (19.3 vs. 10.1%, *P* < 0.001; 3.8 vs. 0.8%, *P* = 0.004, respectively). Data was shown in Table 2.

**Table 2:** Conditions during the stable phase of COPD.

	***Candida* negative group** **(*n*** **= 459)**	***Candida* positive group(*n* = 644)**	***P*-value**
Age (years), median (q1, q3)	78 (69, 84)	78 (69, 83)	0.534
Gender (male/female)	388/71	556/88	0.401
CAT score, median (q1, q3)	22 (15, 27)	24 (19, 30)	<0.001
mMRC score			<0.001
0 (*n*, %)	0, 0%	10, 2%	
1 (*n*, %)	23, 6.7%	36, 7%	
2 (*n*, %)	121, 35.1%	100, 19.6%	
3 (*n*, %)	178, 51.6%	265, 51.9%	
4 (*n*, %)	23, 6.7%	100, 19.6%	
**Post-bronchodilator spirometry**			
FEV_1_(L), median (q1, q3)	0.94 (0.72, 1.20)	0.89 (0.63, 1.23)	0.611
FEV_1_/FVC%, median (q1, q3)	46.32 (41.22, 54.69)	53.80 (42.89, 64.00)	<0.001
**Comorbidities**			
Coronary artery disease (*n*, %)	93, 24%	121, 19.6%	0.093
Left ventricular dysfunction (*n*, %)	39, 10.1%	118, 19.3%	<0.001
Parkinson's disease (*n*, %)	7, 1.8%	6, 1.0%	0.259
Diabetes (*n*, %)	52, 13.4%	108, 17.6%	0.081
Cerebral apoplexy (*n*, %)	20, 5.3%	51, 8.3%	0.083
High blood pressure (*n*, %)	174, 44.5%	252, 40.6%	0.219
OSAS (*n*, %)	3, 0.8%	22, 3.8%	0.004

*COPD, chronic obstructive pulmonary disease; CAT, COPD assessment test; mMRC, modified British research council; FEV1, forced expiratory volume in 1 s; FVC, forced vital capacity; OSAS, obstructive sleep apnea syndrome*.

### Candida Predict Worsening Prognosis of AECOPD

As data shown in Table 3, differences in in-hospital outcome between *Candida* positive group and *Candida* negative group were not statistically significant. Length of hospital stay and hospitalization expense were very close between two groups. The proportion of patients admitted in ICU in *Candida* positive group was higher than that in Candida negative group, but this trend failed to reach statistical significance (7.1 vs. 6.1%, *P* = 0.531). Nevertheless, patients in *Candida* positive group were more likely to have a relapse of AECOPD within 180 days (75.5 vs. 6.6%, *P* < 0.001), and their time to first re-exacerbation was significantly shorter (131.29 ± 143.03 vs. 341.69 ± 87.67 days, *P* < 0.001). Moreover, *Candida* positive group was associated with the higher mortality rate within 1 year (6.9 vs. 0.4%, *P* < 0.001).

**Table 3:** Prognosis of hospitalized AECOPD patients with or without Candida isolation from LTR samples.

	***Candida* negative group** **(*n*** **= 459)**	***Candida* positive group** **(*n*** **= 644)**	***P*-value**
Length of Hospital stay (days)	13 (10, 15)	12 (8, 17)	0.070
Hospitalization expenses (y)	15,953 (12,411; 19,830)	15,499 (10,447; 21,900)	0.715
ICU admission (*n*, %)	28, 6.1%	31, 7.1%	0.531
Length of ICU stay (days)	11 (7, 17)	10 (6, 20)	0.494
mortality within 1 year (*n*, %)	2, 0.4%	27, 6.9%	<0.001
Recurrent AECOPD within 180 days (*n*, %)	22, 6.6%	163, 75.5%	<0.001
Number of recurrences within 1 year, median (q1, q3)	2 (1, 3)	1.5 (1, 3)	0.442
Time to first re-exacerbation	341.69 ± 87.67	131.29 ± 143.03	<0.001

*AECOPD, acute exacerbation of chronic obstructive pulmonary disease; ICU, intensive care unit*.

### Other Factors Related to Prognosis of AECOPD

A significant difference in the prognosis was observed between the *Candida* negative group and the *Candida* positive group. To validate the role of LTR *Candida* isolation on the long-term outcome of hospitalized AECOPD, we performed the statistical analysis of related risk factors of “recurrence of AECOPD within 180 days” and “mortality within 1 year.” Data was shown in Supplementary Table 1.

The higher CAT score and mMRC score during the stable phase were two important risk factors affecting the recurrence of AECOPD within 180 days (OR = 1.073, 95% CI (1.039, 1.108), *P* < 0.001; OR = 1.977, 95% CI (1.475, 2.649), *P* < 0.001, respectively) and mortality within 1 year (OR = 1.142, 95% CI (1.063, 1.226), *P* < 0.001; OR = 2.211, 95% CI (1.250, 3.910), *P* = 0.006, respectively).

Besides, positive *Candida* colonization showed strong relationship with higher recurrence of AECOPD (OR = 43.336, 95% CI (25.456, 73.775), *P* < 0.001) and mortality within 1 year (OR = 16.737, 95% CI (3.954, 70.846), *P* < 0.001). Data showed that high percentage of neutrophils and procalcitonin (PCT) were both risk factors for recurrence of AECOPD within 180 days(OR = 1.017, 95% CI (1.000, 1.033), *P* = 0.044; OR = 1.036, 95% CI (1.008, 1.064), *P* = 0.012, respectively) and mortality within 1 year (OR = 1.042, 95% CI (1.003, 1.082), *P* = 0.032; OR = 1.05, 95% CI (1.028, 1.079), *P* < 0.001). Patients treated with oxygen inhalation also exhibited higher probability of relapse and mortality (OR = 1.992, 95% CI (1.361, 2.915), *P* < 0.001; OR = 3.394, 95% CI (1.457, 7.907), *P* = 0.005, respectively), perhaps due to oxygen inhalation was applied in patients with more serious condition.

### LTR *Candida* Isolation Was Identified as an Independent Risk Factor for AECOPD Relapse Within 180 Days

Considering the results of uni-variate analysis, we recruited variables significant (*P* < 0.05) into the binary logistic regression analysis of recurrent AECOPD within 180 days. Supplementary Table 2 showed that LTR *Candida* colonization, RBC, ESR during AECOPD and the mMRC score during the stable phase were independently associated with recurrent AECOPD within 180 days. Among them, *Candida* colonization status showed the strongest relationship (OR = 1613.620, 95% CI (1.501, 62740.313), *P* < 0.001)

Besides, the binary logistic regression analysis of mortality within 1 year was also analyzed (Supplementary Table 3). The result showed that PCT during AECOPD was an independent risk factor for mortality within 1 year. However, LTR *Candida* colonization may also led to higher mortality within 1 year but this trend failed to reach statistical significance.

## Discussion

In the current study, we found that patients with higher CAT or mMRC score, or patients with severe complications in stable phase of COPD tends to have positive LTR *Candida* isolation, and positive LTR *Candida* significantly worsen the long-term prognosis including recurrence within 180 days and mortality within 1 year. To our knowledge, this is the first study looking into the correlation between LTR *Candida* and the prognosis of hospitalized AECOPD.

Previous studies have suggested that *Candida* colonization might be associated with worse clinical outcomes in critically ill patients. Studies conducted by Delisle et al. (2008) showed a correlation between independent colonization of *Candida albicans* and prolonged hospital stay (59.9 vs. 38.6 days, *P* = 0.006) in ICU patients who fulfilled criteria for a clinical suspicion of ventilator-associated pneumonia (VAP). Azoulay et al. (2006) also observed that patients with *Candida* airway colonization had significantly longer ICU residence time (17(9, 32) vs. 9(5, 17), *P* < 0.001) and hospitalization time (36(19, 74) vs. 22(9, 75), *P* < 0.001). However, extended hospitalization and ICU residence time of *Candida* positive group was not observed in our study, and we believed that it was mainly due to different research populations. Previous studies focused on VAP and ICU-acquired pneumonia patients, while our study enrolled patients with AECOPD. Therefore, we suggest that whether there is a correlation between *Candida* colonization and prolonged hospital stay or ICU residence time could mainly depend on the primary disease.

Mortality is an important indicator of disease prognosis. We observed a significant trend of higher mortality in Candida positive group. It was in accordance with previous studies which found that there was an independent correlation between colonization of airway *Candida* and a higher hospital mortality (Delisle et al., 2008; Williamson et al., 2011; Hamet et al., 2012). There were also other researches indicated that *Candida* colonization was not related to an increased in-hospital mortality (Wood et al., 2006; Roux et al., 2009b; Terraneo et al., 2016). However, all these studies were performed in hospitalized or ICU patients with VAP or ICU-acquired pneumonia. Whether there is a correlation between positive LTR *Candida* and mortality of AECOPD needs to be further studied.

Recurrence of AECOPD was the main objective of our research. To our knowledge, the clinical relevance of LTR *Candida* isolation and recurrence of AECOPD has not been established. Our research exhibited that patients in positive *Candida* group were more likely to have a relapse of AECOPD within 180 days. Binary multi-variate regression analysis approved that positive *Candida* was independently associated with recurrent AECOPD within 180 days. Besides, that patients in *Candida* group suffered higher death rate.

In theory, LTR *Candida* may cause detrimental effects in different aspects. Firstly, *Candida* colonized in the respiratory tract would further adhere to, invade and infect under certain conditions. It was considered that *Candida* colonization was the precursor risk factor of *Candida* infection, and may be the risk factor and predictive index of deep *Candida* infection (Eggimann et al., 2003).

Secondly, *Candida* could interact with local pathogenic bacteria, affect the occurrence and development of bacterial infection through direct contact or indirect regulation (Ricard and Roux, 2012; Mear et al., 2013), changing the pathogenic process of each other (Shirtliff et al., 2009; De Pascale and Antonelli, 2014). Thus, patients with *Candida* colonization had a greater chance to acquire pneumonia caused by bacteria infection. A retrospective study in China found that respiratory *Candida* colonization could increase the risk of bacterial VAP in patients with MV, and the reason may be that *Candida* colonized in human respiratory tract increased the chance of bacterial infection by interacting with pathogenic bacteria (Dong-xing et al., 2016). A few retrospective studies have shown that airway *Candida* colonization could promote VAP development, especially when it was caused by *Pseudomonas aeruginosa* (Azoulay et al., 2006; Roux et al., 2009a). Besides, it has been shown that *Staphylococcus aureus* could change into resistant phenotype when growing within *Candida* spp. (Harriott and Noverr, 2009). *Candida* may also attributed to the formation of biofilm through interactions with other pathogens such as *Staphylococcus aureus*, resulting in a more effective pattern for pathogens to escape from host immunity (Roux et al., 2009a; Morales and Hogan, 2010; Peters et al., 2010). All these findings suggested that *Candida* colonization within the respiratory tract could facilitate bacterial growth and defense against antibiotics. For patients with severe COPD, increased bacteria burden would lead to the onset of exacerbation.

Thirdly, it was also proved that *Candida* could cause allergic bronchopulmonary mycosis (Chowdhary et al., 2014), so the sensitization of colonized *Candida* could lead to respiratory symptoms.

All these researches may explain the higher rate of recurrence of AECOPD in LTR *Candida* positive patients.

Antifungal treatment is not routinely recommended in case of positive *Candida* isolation from LTR samples, because pneumonia is rarely caused by this fungal species (Garnacho-Montero et al., 2013). Studies had also shown that antifungal therapy was not associated with better outcomes of patients with LTR *Candida* spp. isolation (Wood et al., 2006; Terraneo et al., 2016). Considering the data in the current study, LTR *Candida* isolation in patients with hospitalized AECOPD should be monitored more carefully and frequently, and evaluation the individualized need for de-colonization therapy is necessary for optimal outcome. More importantly, study on the mechanism how *Candida* interact with other pathogens as well as human immune system and how this interaction leads to AECOPD are required, with the purpose of finding new interventions.

Our study has several limitations. Firstly, it is a retrospective study, therefore the correlation between the variables could be proven but could not be explained as causal relationship. Secondly, subjective bias cannot be avoided in some ways. We plan to verify the results in prospective studies in the future. Thirdly, we were not able to distinguish whether the patients were colonized with *Candida* before AECOPD or secondary to the treatment of AECOPD. Fourthly, we could not distinguish if it was *Candida* infection or *Candida* colonization, either, which is a common problem in similar researches (Olaechea et al., 2004; Azoulay et al., 2006).

In spite of these limitations, the results can lead to the convincing conclusion that the LTR *Candida* isolation was associated with worse outcomes of AECOPD patients. There was an independent association between positive LTR *Candida* with exacerbation relapse within 180 days of hospitalized AECOPD, and it might contribute to an increased 1 year mortality. Besides, it is suggested that the risk of *Candida* positive in patients with severe symptoms is higher, and the detection should be strengthened. Clinical research with prospective design and adequate sample size are required to validate this relationship and explore specific intervention strategy to improve patients' outcome. Attention should be paid to the impact of *Candida* on AECOPD patients in clinic practice.

## Data Availability Statement

The original contributions presented in the study are included in the article/Supplementary Material, further inquiries can be directed to the corresponding authors.

## Ethics Statement

The ethics committee of Ruijin Hospital approved the study (Number: 2017-108). This study was registered on Chinese Clinical Trial Registry (Registration Number: ChiCTR-RRC-17014078). Informed consent was waived because the study was retrospective.

## Author Contributions

Y-hZ analyzed data and wrote the paper. W-qW was in charge of monitoring the study progress and collecting data. The following authors contributed to data collection: Q-jC, BL, F-yZ, X-yJ, Jq-H, H-yL, Z-yB, Z-jJ, G-fW, X-wG, HS, and J-fX. JZ and J-mQ designed the study. All authors contributed to the article and approved the submitted version.

## Conflict of Interest

The authors declare that the research was conducted in the absence of any commercial or financial relationships that could be construed as a potential conflict of interest.
