# Supplementary material for: Candida in Lower Respiratory Tract Increases the Frequency of Acute Exacerbation of Chronic Obstructive Pulmonary Disease: A Retrospective Case-Control Study
Source: Front Cell Infect Microbiol. 2020 Sep 30;10:538005. doi: 10.3389/fcimb.2020.538005 (PMC7561360; doi:10.3389/fcimb.2020.538005)
Supplement: Supplementary file 1 [file Data_Sheet_1.docx]

Supplemental Table 1. Uni-variate analysis of factors related to prognosis of AECOPD

| Variables | Recurrent AECOPD  within 180 days | | | Mortality within 1 year | | |
| --- | --- | --- | --- | --- | --- | --- |
|  | OR | 95% CI | P value | OR | 95% CI | P value |
| **Conditions during stable phase of COPD** |  |  |  |  |  |  |
| Age | 1.009 | (0.990, 1.029) | 0.344 | 1.049 | (1.001, 1.099) | 0.046 |
| Sex | 1.048 | (0.628, 1.749) | 0.858 | 1.207 | (0.452, 3.224) | 0.708 |
| CAT score | 1.073 | (1.039, 1.108) | <0.001 | 1.142 | (1.063, 1.226) | <0.001 |
| mMRC score | 1.977 | (1.475, 2.649) | <0.001 | 2.211 | (1.250, 3.910) | 0.006 |
| GOLD | 1.082 | (0.727, 1.610) | 0.699 | 0.796 | (0.240, 2.643) | 0.709 |
| FEV_1_ | 1.621 | (0.692, 3.796) | 0.266 | 6.780 | (0.916, 50.166) | 0.061 |
| FVC | 0.682 | (0.406, 1.144) | 0.147 | 1.599 | (0.367, 6.973) | 0.532 |
| FEV_1_/FVC | 1.005 | (0.996, 1.014) | 0.286 | 1.000 | (0.954, 1.049) | 0.986 |
| Left ventricular dysfunction | 1.718 | (1.031, 2.861) | 0.038 | 1.656 | (0.653, 4.197) | 0.288 |
| Coronary disease | 0.813 | (0.526, 1.256) | 0.351 | 1.933 | (0.896, 4.171) | 0.093 |
| OSAS | 3.612 | (0.655, 19.931) | 0.141 | 0.000 | / | 0.999 |
| Diabetes | 1.527 | (0.935, 2.492) | 0.091 | 1.695 | (0.708, 4.060) | 0.236 |
| Apoplexy | 1.205 | (0.607, 2.391) | 0.593 | 2.407 | (0.803, 7.215) | 0.117 |
| **Conditions of AECOPD** |  |  |  |  |  |  |
| Fever | 1.042 | (0.696, 1.561) | 0.840 | 1.070 | (0.480, 2.385) | 0.868 |
| Candida | 43.336 | (25.456, 73.775) | <0.001 | 16.737 | (3.954, 70.846) | <0.001 |
| RBC | 0.706 | (0.522, 0.956) | 0.024 | 0.583 | (0.339, 1.003) | 0.051 |
| Hb | 0.995 | (0.986, 1.004) | 0.260 | 0.984 | (0.968, 1.000) | 0.046 |
| PLT | 0.998 | (0.996, 1.001) | 0.140 | 1.002 | (0.977, 1.006) | 0.439 |
| WBC | 0.985 | (0.939, 1.032) | 0.519 | 1.017 | (0.933, 1.108) | 0.705 |
| N% | 1.017 | (1.000, 1.033) | 0.044 | 1.042 | (1.003, 1.082) | 0.032 |
| E% | 0.968 | (0.917, 1.002) | 0.246 | 1.03 | (0.977, 1.095) | 0.247 |
| CRP | 0.997 | (0.993, 1.002) | 0.211 | 0.992 | (0.982, 1.003) | 0.146 |
| PCT | 1.036 | (1.008, 1.064) | 0.012 | 1.053 | (1.028, 1.079) | <0.001 |
| ESR | 1.010 | (1.001, 1.019) | 0.022 | 1.005 | (0.993, 1.018) | 0.425 |
| **CT examination** |  |  |  |  |  |  |
| patchy | 1.075 | (0.723, 1.597) | 0.721 | 1.821 | (0.704, 4.709) | 0.217 |
| consolidation | 2.613 | (1.221, 5.593) | 0.013 | 3.670 | (1.177, 11.447) | 0.025 |
| Multi-lobe lesions | 1.464 | (0.936, 2.291) | 0.095 | 2.679 | (1.140, 6.296) | 0.024 |
| Ph | 0.110 | (0.007, 1.804) | 0.122 | 0.513 | (0.001, 184.293) | 0.824 |
| PaO2 | 0.998 | (0.993, 1.002) | 0.309 | 1.001 | (0.992, 1.011) | 0.808 |
| PaCO2 | 1.006 | (0.997, 1.015) | 0.176 | 1.012 | (0.996, 1.029) | 0.147 |
| BE | 0.967 | (0.935, 0.999) | 0.045 | 1.013 | (0.961, 1.067) | 0.640 |
| HCO3- | 0.974 | (0.944, 1.005) | 0.105 | 1.056 | (0.999, 1.115) | 0.055 |
| Oxygen inhalation | 1.992 | (1.361, 2.915) | <0.001 | 3.394 | (1.457, 7.907) | 0.005 |
| Length of hospital stay (days) | 1.011 | (0.987, 1.035) | 0.368 | 1.056 | (1.028, 1.084) | <0.001 |
| ICU admission | 0.651 | (0.275, 1.541) | 0.329 | 1.379 | (0.315, 6.047) | 0.670 |
| Length of ICU stay | 0.991 | (0.960, 1.022) | 0.558 | 0.970 | (0.834, 1.128) | 0.693 |
| Hospitalization expense | 1.000 | (1.000, 1.000) | 0.194 | 1.000 | (1.000, 1.000) | <0.001 |

Abbreviations: COPD: chronic obstructive pulmonary disease; CAT: COPD assessment test; mMRC: modified British research council; GOLD: global initiative for chronic obstructive lung disease; FEV1: forced expiratory volume in 1 second; FVC: forced vital capacity; OSAS: obstructive sleep apnea syndrome; RBC: red blood cell; Hb: hemoglobin; PLT: blood platelet; WBC: white blood cell; N: neutrophil; E: eosinophil; CRP: C-reaction protein; PCT: procalcitonin; ESR: erythrocyte sedimentation rate; CT: computed tomography; BE: base residue; ICU: intensive care unit.

Supplemental Table 2. Binary logistic regression analysis of factors associated with recurrent AECOPD within 180 days

|  | Recurrent AECOPD within 180 days | |
| --- | --- | --- |
|  | OR (95%CI) | P value |
| **The CAT score** | **1.237(1.042, 1.469)** | **0.015** |
| The mMRC score |  | 0.202 |
| mMRC score 1 | 0.321(0.019, 5.506) | 0.433 |
| mMRC score 2 | 9.862(0.356, 272.947) | 0.177 |
| mMRC score 3-4 | 1.650(0.178, 15.301) | 0.659 |
| Left ventricular dysfunction | 0.311(0.029, 3.333) | 0.334 |
| **Candida** | **178.642(23.549, 1355.190)** | **<0.001** |
| **RBC** | **0.119(0.017, 0.822)** | **0.031** |
| N% | 1.010(0.935, 1.091) | 0.796 |
| **ESR** | **0.931(0.836, 0.977)** | **0.004** |
| BE | 0.976(0.848, 1.124) | 0.737 |
| Consolidation | 2.370(0.032, 176.114) | 0.695 |
| Oxygen inhalation | 2.438(0.511, 11.637) | 0.264 |

Abbreviations: AECOPD: acute exacerbation of chronic obstructive pulmonary disease; CAT: COPD assessment test; mMRC: modified British research council; PCT: procalcitonin; RBC: red blood cell; N: neutrophil; ESR: erythrocyte sedimentation rate; BE: base residue.

Supplemental Table 3. Binary logistic regression analysis of factors associated with Mortality within 1 year

|  | Mortality within 1 year | |
| --- | --- | --- |
|  | OR (95%CI) | P value |
| age | 1.200(0.994, 1.448) | 0.058 |
| The CAT score | 1.271(0.967, 1.672) | 0.086 |
| The mMRC score |  | 0.529 |
| mMRC score 1 | 0.000 | 0.999 |
| mMRC score 2 | 47.555(0.222, 10177.750) | 0.158 |
| mMRC score 3-4 | 5.950(0.125, 282.291) | 0.365 |
| LTR Candida isolation | 2.764(0.334, 22.582) | 0.346 |
| **PCT** | **1.093(1.017, 1.174)** | **0.016** |
| Hb | 1.049(0.980, 1.123) | 0.167 |
| N% | 1.019(0.922, 1.126) | 0.711 |
| Consolidation on radiological investigation | 5.433(0.380, 77.787) | 0.213 |
| Multi-lobe lesions on radiological investigation | 1.517(0.168, 13.659) | 0.710 |
| Oxygen therapy during AECOPD | 1.363(0.154, 12.053) | 0.781 |
| Length of hospital stay | 0.953(0.743, 1.224) | 0.708 |
| Hospitalization expense | 1.000(1.000, 1.000) | 0.102 |

Abbreviations: AECOPD: acute exacerbation of chronic obstructive pulmonary disease; LTR: lower respiratory tract; CAT: COPD assessment test; mMRC: modified British research council; PCT: procalcitonin; Hb: hemoglobin; N: neutrophil.
